# Cellphone separation modulates the effects of working memory load on ex-Gaussian parameters of choice reaction time

**DOI:** 10.1186/s41235-025-00684-9

**Published:** 2025-10-22

**Authors:** Michael Gazzanigo, Alexa Quesnel, Catalina Roldan, Xiao Yang

**Affiliations:** Department of Psychology, Old Dominion University (ODU), 346G Mills Godwin Life Sciences Building, Norfolk, VA 23529 USA

**Keywords:** Cellphone separation, Working memory, Choice reaction time, Ex-Gaussian modeling

## Abstract

Cognitive effects of cellphone dependency among young adults have garnered increasing research attention. While cellphones have been identified as a distractor in daily tasks, related psychological processes remain unclear. As a potential mechanism underlying those effects of cellphones, excessive working memory (WM) load has not yet been well examined. Our study investigated the effects of the mental representation of cellphone separation on WM. Seventy-five participants (*M*_*age*_ = 21.3 years; 55 females, 20 males) were assigned into three groups: the cued separation, natural separation, or control group, and completed a block of choice reaction time (CRT) task, and a dual-task block: the CRT and a concurrent WM task. CRT performance was analyzed using the ex-Gaussian model, providing the parameters *μ* and *τ* to reflect lower-order processing and top-down control, respectively. Results showed that WM load reduced cognitive performance, with the cued separation group exhibiting the largest performance impairments, and ex-Gaussian *μ* and *τ* were sensitive to WM load and cellphone separation. Our findings suggest that the mental representation of cellphone separation, especially when cued, depletes cognitive resources, and impairs executive functions, which highlight the need for strategies to mitigate the cognitive costs of cellphone dependency, particularly in high-stakes applied contexts.

## Introduction

Cellphone use has become an integral aspect of most people’s everyday life. Two out of every three people were frequent cellphone users in 2024, and the global population of cellphone users was predicted to surpass six billion by 2027 (Turner, 2025). Cellphones and other mobile devices enhance communication, productivity, and efficiency in obtaining information, inducing an increased dependency on these devices among people in technological societies (McElroy & Young, 2024; Wilmer et al., 2017). Resultantly, the compulsion to use these pervasive devices distracts the users and influences their performance during daily tasks (Chu et al., 2021; Liu et al., 2022; Rosen et al., 2013; Wilmer et al., 2017). For example, using cellphones while driving drastically increases the risk of car crash (Chee et al., 2021; Zhang et al., 2024). This effect is further evidenced by lower academic performance in colleges consequent of cellphone dependency (Levine et al., 2007; Liu et al., 2024).

Young people are particularly susceptible to the negative psychological effects associated with cellphone dependency. Compared to other age groups, young adults adopt new technologies faster and use those technologies more often (Chu et al., 2021; Levine et al., 2007; Thornton et al., 2014). As a result, this population is more likely to develop symptomatic cellphone dependency leading to poor sleep quality, low levels of health behavior and social interaction, and mental health problems in this population (for reviews, see Girela-Serrano et al., 2024; Notara et al., 2021; Wacks & Weinstein, 2021). Moreover, young individuals’ overreliance on cellphones in communication may give rise to *nomophobia*, a condition identified as anxiety or discomfort emerging from being without cellphones or even not using the device (Clayton et al., 2015; Mendoza et al., 2018). In conjunction, cellphone dependency and cellphone separation anxiety have adverse behavioral and health outcomes in young adults.

The negative effects of cellphone use can be explained by impairments of *executive functions* (EFs). EFs refer to a set of effortful cognitive processes that monitor and control other mental activities (Baddeley, 1986; Dimond, 2013). EFs underlie the engagement and maintenance of health behavior (Allan et al., 2016; Gray-Burrows et al., 2019), and EF impairment has been linked to impulsive behavior and addiction (Bickel et al., 2012; Domínguez-Salas et al., 2016). Further, EFs play an important role in emotion regulation and provide top-down resources to fortify an individual against psychological disorders (Beauchaine & Thayer, 2015; Friedman & Thayer, 1998; Kemp et al., 2010). Among various components of EFs, working memory (WM) is critical in coordinating goal-directed behavior but is particularly vulnerable to distractions (Baddeley, 1986; Shallice & Burgess, 1993). Increased levels of WM load reduce performance on concurrent cognitive tasks (King & Schaefer, 2011; Yang et al., 2021, 2024). Additionally, according to the attentional control theory, when compensatory strategies are used, anxiety may not reduce the effectiveness of cognitive performance but may increase effort and heighten cognitive load (Eysenck et al., 2007).

Consistent with the line of research on WM and behavioral performance, several studies have examined the effects of cellphones on cognitive processes among young adults. It has been reported that attention resources were automatically and involuntarily allocated to processing the sensory stimuli of cellphone notifications (Roye et al., 2007; Stothart et al., 2015). This detrimental effect of cellphones on cognitive resources is also exhibited in classrooms in the form of impaired recollection of lecture content among college students (Lee et al., 2020; Mendoza et al., 2018), implying WM as a potential mediator in the cognitive impairment related to cellphones (Clayton et al., 2015; Ge et al., 2023; Liebherr et al., 2020; Wilmer et al., 2017).

Laboratory studies have further clarified potential mechanisms of cognitive effects of cellphones. Cellphones influence WM through their mental representations rather than actual interactions between the device and the users. The mere presence of a cellphone decreased performance during a battery of EF tasks (Thornton et al., 2014) and reduced the engagement of social interaction (Przybylski & Weinstein, 2013). Although the cellphone’s state (on or off) did not influence available cognitive resources, the factors determining the availability of cellphones modulated WM resources and cognitive performance (Ward et al., 2017). The negative effects on cognition related to the mental representations of available cellphones have been replicated in studies using a spatial visual search (Ito & Kawahara, 2017), a letter recognition-sensory detection dual task (Liu et al., 2022), attention tasks (Canale et al., 2019; Skowronek et al., 2023), memory assessments (Tanil & Yong, 2020), and a reasoning task (Schwaiger & Tahir, 2020). The deteriorating effect of cellphone on cognition might also be explained by mental representations of a reward cue (i.e., cellphones; Ward et al., 2017). The associations of cellphones with distraction and rewards of information access and social connection are likely established from daily use of the mobile device (Hartanto & Yang, 2016; Ward et al., 2017).

Contrasting the accumulating evidence of the negative cognitive effects of cellphones, null findings were also reported. A few recent studies failed to replicate the effects of cellphones on memory and EF performance (e.g., Hartmann et al., 2020; Ruiz Pardo & Minda, 2022). In a recent meta-analysis, Hartanto et al. (2024) found that the mere presence of cellphones did not show had no significant decreasing effect on cognitive outcomes and suggested that isolation from cellphones may not improve productivity and performance in applied settings. Mixed findings in this research area call for a closer examination on psychological constructs and experimental manipulations. One possible factor that influences results of a study is whether the manipulation induces nomophobia. Although the presence of a cellphone may serve as a distractor, cellphone separation could lead to anxiety that also impairs WM (Hartanto & Yang, 2016). Therefore, the most harmful effects may be generated by an active mental representation of a cellphone and a physical separation of the device, compared to the mere presence or separation. Unfortunately, this possibility has not been empirically studied.

Few prior studies have directly manipulated WM load, producing another research gap to be remedied. The dual task paradigm is an approach implemented in the minority of studies that manipulated WM load, in which participants complete a primary cognitive task and a secondary task simultaneously (e.g., King & Schaefer, 2011; Klauer et al., 2014; Yang et al., 2021, 2024); the secondary task effectively decreases available WM resources and causes poor cognitive performance on the primary task. Given that the mental representation of cellphones occupies the limited WM resources, the presence of a cellphone and the activation of nomophobia will enlarge the negative effect of WM load on the performance of the primary task. Liu et al. (2022) used a dual task to investigate the effects of cellphone presence on cognitive performance; however, the secondary tasks were perceptual tasks that did not reflect the recruitment of WM resources. Therefore, compared to the cognitive tasks used in the previous studies (e.g., visual search and attention tasks), the dual task paradigm with manipulations of WM load can more directly and causally investigate the association between cellphones and WM.

In addition to the dual task paradigm, reaction times (RTs) have served as an objective indicator of cognitive processes (Saltzman & Garner, 1948; Sanders, 1980). Although RTs alone primarily reflect sensorimotor processing, more sophisticated analyses of RT data may separate top-down control from lower level sensory-perceptual processing (Heathcote et al., 1991; Spieler et al., 1996). The commonness of skewed distributions in RT data negates the values of traditional metrics of RT (e.g., mean and median RT) that are influenced by extreme RT trials and fail to fully capture the trial-by-trial variability in RTs (Heathcote et al., 1991; Yang et al., 2023). This sizable limitation of the traditional measurements has motivated many researchers to incorporate the ex-Gaussian parameters into their data processing procedures in recent years for more precise analysis (Whelan, 2008).

The ex-Gaussian model is appropriate for the natural distribution of RT data and isolates exceedingly slow responses (the exponential tail of the RT distribution) from faster trials in the Gaussian portion of the RT distribution (Steinhauser & Hübner, 2007; Whelan, 2008). This method provided researchers with different components of an ex-Gaussian distribution to indicate separate aspects of behavioral performance. Specifically, the ex-Gaussian parameters *mu* and *sigma* (*μ* and *σ*), representing the mean and standard deviation of the Gaussian component of the skewed RT distribution, are thought to indicate low-order sensory-perceptual processing; the parameter tau (*τ*) describes the very slow RTs within the exponential tail portion of the skewed RT distribution and putatively reflects top-down control processes (Gmehlin et al., 2016; Leth-Steensen et al., 2000). Further, the ex-Gaussian *τ* has been found sensitive to the increased WM load induced by the concurrent secondary task in the dual task paradigm (Yang et al., 2023, 2024).

## Method

### Participants

Seventy-five participants (*M*_*age*_ = 21.30 years; *SD* = 4.98 years; 55 females, 20 males) were recruited from psychology courses at Old Dominion University (ODU). Initially, G*Power version 3.1 was used to estimate the required sample size. Specifically, in the priori power analysis, the significance level (*α*) and power (1-*β*) were set as 0.05 and 0.8, respectively; and, based on the previous studies of cellphone separation and dependence (Clayton et al., 2015; Lee et al., 2020), a small effect size (0.38) was assumed, which resulted in a sample size of 69 to detect a desired effect. The additional participants were recruited to prevent the impact of attrition on data analyses. Due to the limitations of using Cohen’s definitions of effect sizes (Correll et al., 2020), when the initial participant recruitment was completed, a post-hoc power analysis was conducted to confirm the power of the study with the sample size and collected data. A web-based program GLIMMPSE (Guo et al., 2013) was used to calculate the power of repeated measures ANOVA with an interaction between within-subject and between-subjects factors (see later sections for the experimental design). The results of the post-hoc power analysis indicated a power of 0.82 at the significance level of 0.05 (*α*). Therefore, no additional participants were recruited after the initially planned recruitment.

All participants were non-smokers and reported no auditory, visual, or mental health issues. Due to the usage of colorful stimuli, the study was screened for any type of color blindness using a single self-reported item (“*Do you have any known color vision deficiency? If yes, what type?*”) on a health history questionnaire. Moreover, the genders of all participants were biological binary, and no participants were identified as *other gender*. Approval for the study was obtained from the ODU Institutional Review Board (the approved protocol ID: 1,979,162–3); all participants provided verbal and written consent prior to participation. Two participants were excluded due to equipment failures, and two other participants did not complete the experimental tasks and were also excluded from data analysis, resulting in a final sample of 71 participants (*M*_*age*_ = 21.15 years; *SD* = 5.06 years; 53 females, 18 males).

### Materials

The State-Trait Inventory (STAI; Spielberger et al., 1983) was used to assess the level of state anxiety among participants. The State Anxiety Inventory subscale of the STAI includes 20 items on a 4-point Likert scale (Spielberger et al., 1983). Previous investigations have established that the STAI has good internal consistency and test–retest reliability (Barnes et al., 2002). In the present study, only the State Anxiety Inventory subscale was estimated at Cronbach’s α of 0.78 and 0.76 before and after the cognitive tasks, respectively, indicating an acceptable internal reliability.

The Media and Technology Usage and Attitudes Scale (MTUAS) was used to assess participants’ cellphone usage levels. The MTUAS is a self-report questionnaire that includes 60 items on a 10-point Likert scale used to assess the frequency of the technology usage behaviors and 18 items on a 5-point Likert scale that measured the attitudes users have pertaining to technology usage, which has shown good internal consistency and test–retest reliability (Rosen et al., 2013). Given the purpose of the present study, the cellphone usage subscale of the MTUAS was used, which measures technology use behaviors such as social media use, checking emails, texting, calling, etc. that would prompt cellphone users to check their phone (Rosen et al., 2013). Higher scores on this subscale indicate higher levels of usage. In the current study, the estimated Cronbach’s α of the cell phone usage subscale was 0.80.

The NASA Task Load Index (NASA-TLX) was used to assess subjective workload during the cognitive tasks. The NASA-TLX is a self-rated questionnaire of perceived workload and includes six items on a 7-point scale. In the computer version of the NASA-TLX, there are increments of low, medium, and high estimates for each point, which generate a 21-point Likert scale to assess dimensions of workload: mental demand, physical demand, temporal demand, performance, perceived effort, and frustration, respectively (Hart & Staveland, 1988). The NASA-TLX has been used in laboratory and real-life settings and indicate a good test–retest reliability (Hart & Staveland, 1988) and acceptable to good internal consistencies (Zheng et al., 2012). Subjective workload during WM tasks is primarily associated with ratings of mental demand (Seidman et al., 2023; Westbrook et al., 2023). Given the focus on subjective experiences of WM load, the single score of the first item of NASA-TLX on mental demand “*How mentally demanding (e.g., thinking, deciding, calculating, remembering, looking, searching, *etc*.) was the task*?” was analyzed in the current study. While the NASA-TLX is usually used to assess task-induced workload after single tasks, we used paired comparisons of TLX total scores for the two task conditions. To minimize the accumulating effect on subjective workload by multiple assessments, the order of the task conditions was randomly counterbalanced across participants (see the later section).

### Design and tasks

A split-plot design was used in the study. The participants were randomly assigned into one of the three groups: the cued separation group, the natural separation group, and the control group (see the detailing of the groups in the procedure section; Skowronek et al., 2023; Ward et al., 2017). Group membership was the between-subjects factor. Moreover, each participant completed two blocks of a choice RT task, with and without a concurrent WM task. WM load level was the within-subject factor.

**Fig. 1:**
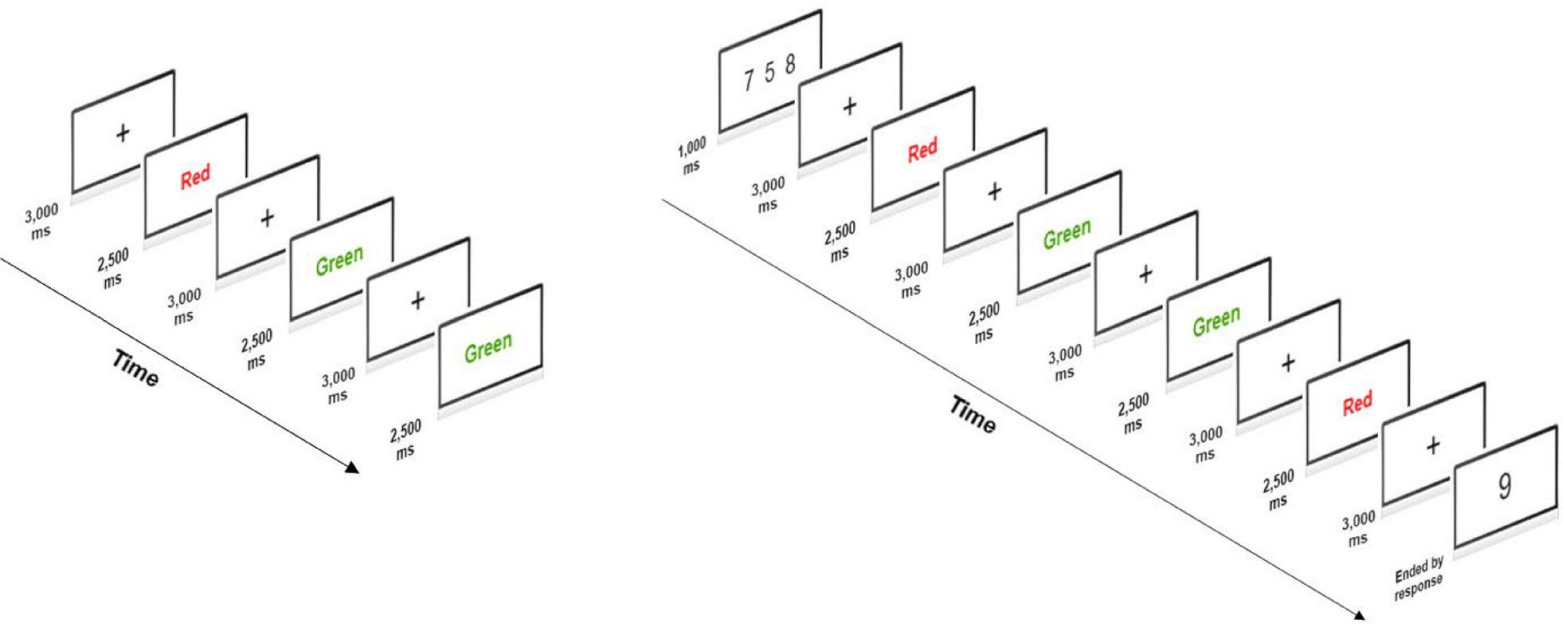
Stimulus Presentation in the Choice Reaction Time (CRT) Task and the Dual-Task Paradigm. The left panel shows the trials in the CRT task block; the right panel shows the CRT task and the concurrent working memory task

In the choice RT task, the words “Red” and “Green” were presented in the middle of a computer screen serving as the RT stimuli. In each RT trial, the participant would press the left arrow key on a computer’s keyboard in response to “Red” and the right arrow key in response to “Green.” The RT stimulus was terminated by a detected response or after 2,500 ms (ms) if no key pressing was detected. The intertrial interval was 3,000 ms (see Fig. 1). The visual stimuli were presented on a 61-cm diagonal wide computer screen that was located 50 cm in front of the subject, using the E-Prime 3.0 software (Psychology Software Tools, Pittsburgh, PA).

The Sternberg memory task was used to manipulate WM load, in which participants were asked to memorize three digits. In a WM trial, digits were displayed on the screen for 1,000 ms, after which participants completed four choice RT trials. The probe digit was presented after the choice RT trials and participants indicated whether a probe digit was among the three digits by pressing “Z” (to confirm the probe) or “X” (to reject the probe) on a computer keyboard. The duration of memory maintenance periods was approximately 20,000 ms (see Fig. 1).

There was a total of 200 choice RT trials, including a block of 100 CRT trials without enhanced WM load and a block of 100 RT trials with WM load. Moreover, 25 Sternberg memory trials were delivered in the enhanced WM load block. The order of the two blocks was randomized across participants.

### Procedure

Upon arrival at the laboratory, informed consent was obtained from all participants. Participants then completed a health history questionnaire, the MTUAS and the State Anxiety Inventory. Afterward, participants were randomly assigned into the three groups. Specifically, in *the cued cellphone separation group*, a cellphone that did not belong to the participant was placed on the desk before participants began the experimental tasks and they were also asked to move their cellphones to another room. In *the natural separation group*, participants were only asked to move their cellphones to another room. In *the control group*, participants had their phones with them during the entire experiment and did not receive any instructions regarding their cellphones.

Following the group assignment, participants were comfortably seated in the laboratory as the choice RT task and the WM task were introduced to the participants. After ten practice trials, participants completed a block of 100 CRT trials with no enhanced WM load and a block of 100 trials of the dual task (the CRT and enhanced WM load tasks). The order of the two blocks was randomized across participants. Participants took a 2-min break between the two CRT blocks and rated their perceived workload using NASA TLX after each block of the CRT.

At the end of the second block, participants completed the State Anxiety Inventory again and were then asked to sit still and remain quiet for 2 min as the recovery period, after which participants were thanked and informed about the purpose of this study.

### Data reduction

Cognitive performance was assessed through response accuracy and RTs. Response accuracy was determined as the percentage of correct responses in each block. RTs were analyzed using ex-Gaussian modeling with trials shorter than 100 ms excluded from analyses—this constituted less than 0.1% of all RT trials for each block within a participant. Note that the outlier exclusion procedure followed Miller’s (2023) recommendation that wide cutoffs should be used to allow reasonable RT trials. In the present study, with the 2,500 ms response window, the cutoffs to include a RT trial was set as 100 ms—2,500 ms, which was also consistent with the feature of long tail of the RT distribution (the parameter *τ*; Luce, 1986) and Ratcliff’s (1993) guideline. The ex-Gaussian model was applied to the RT distribution for each condition, utilizing the “retimes” package in R statistical software. Parameters *μ*, *σ*, and *τ* were estimated through bootstrap resampling (1000 iterations) to ensure model robustness.

### Data analysis

Demographic and self-report data across the three groups were compared using one-way analysis of variance (ANOVA) or χ^2^ tests as appropriate. To explore the effects of concurrent WM load and cellphone separation groups on behavioral performance, participants’ TLX mental demand scores, response accuracy, and ex-Gaussian parameters were analyzed using 2 (WM load) × 3 (Group) repeated measures ANOVA. Significant main effects and interactions were further examined with two-tailed paired t-tests, adjusted using the Bonferroni correction. Statistical significance was established with an alpha level of 0.05 and effect sizes were estimated using partial eta-squared (*η*^*2*^_*p*_).

## Results

### Descriptive statistics and manipulation check

Descriptive statistics are presented in Table 1. There were no significant differences in demographic variables, cellphone usage, or state anxiety among the three groups (see Table 1), indicating that the groups were equivalent at baseline. Additionally, behavioral performance was similar across the groups (see Table 1).

**Table 1:** Descriptive Statistics of the Samples

Variable	Cued separation (*n* = 23)	Natural separation (*n* = 23)	Controls (*n* = 25)	*F* or* χ*^*2*^ statistics	
Female (*n,* %)	17 (73.9%)	18 (78.3%)	18 (72.0%)	0.26	
Age (M years, *SD*)	20.1 (2.7)	22.1 (7.0)	21.2 (4.6)	0.82	
Cell phone usage (M, *SD*)	102.4 (15.9)	98.9 (15.2)	95.0 (15.4)	1.34	
State anxiety (M, *SD*)					
Baseline	30.9 (7.0)	31.4 (9.7)	34.2 (7.6)	1.18	
After CRT tasks	38.3 (12.5)	38.9 (12.3)	37.9 (8.2)	0.05	
Mental demand (M, *SD*)					
CRT task alone	6.3 (5.5)	6.9 (4.3)	7.2 (6.0)	0.17	
Dual task	10.5 (7.2)	9.9 (5.2)	10.9 (6.3)	0.14	
∆TLX	4.1 (4.7)	3.0 (3.7)	3.6 (4.0)	0.41	
Response accuracy (M%, *SD*)					
CRT task alone	94.4 (14.0)	96.1 (10.8)	97.0 (4.9)	0.39	
Dual task	97.0 (8.9)	97.8 (2.0)	95.9 (8.9)	0.61	
∆Accuracy	2.6 (1.4)	1.7 (1.1)	−1.1 (2.1)	0.56	
Ex-Gaussian mu (M ms, *SD*)					
CRT task alone	426.3 (100.8)	461.9 (115.8)	462.0 (105.3)	0.84	
Dual task	488.5 (84.3)	497.6 (109.4)	491.5 (79.5)	0.06	
∆mu	62.2 (77.7)	35.7 (73.1)	29.5 (92.8)	0.40	
Ex-Gaussian sigma (M ms, *SD*)					
CRT task alone	60.8 (25.9)	66.7 (35.9)	84.9 (47.2)	2.69	
Dual task	77.2 (35.1)	78.1 (32.6)	80.0 (23.5)	0.06	
∆sigma	16.4 (19.3)	11.4 (26.1)	−4.8 (30.0)	0.04	
Ex-Gaussian tau (M ms, *SD*)					
CRT task alone	134.0 (46.5)	178.1 (103.2)	162.3 (81.7)	1.77	
Dual task		290.2 (126.5)	242.9 (124.9)	261.0 (138.1)	0.77
∆tau		156.2 (112.7)	64.7 (75.9)	98.7 (101.2)	0.01
WM accuracy (M %, *SD*)	91.6 (8.3)	92.7 (8.1)	89.7 (10.5)	0.68	
WM response time (M ms, *SD*)	2181.1 (704.9)	2126.4 (961.8)	2170.2 (1057.3)	0.02	

*CRT* Choice reaction time; *WM* Working memory. State anxiety was assessed by the Spielberger State Anxiety Inventory before and after the experimental tasks; Cellphone usage was assessed by the Cellphone Usage subscale of the Media and Technology Usage and Attitudes Scale; Mental demand was measured by the first item of the NASA TLX scale “*How mentally demanding (e.g., thinking, deciding, calculating, remembering, looking, searching, *etc*.) was the task*?”; Dual task involved the CRT and a concurrent Sternberg WM task. ∆ scores indicate the differences between the CRT alone and the dual task conditions (*Dual Task*–*CRT alone*)

A repeated measures ANOVA of state anxiety revealed a significant main effect of cognitive tasks, *F*(1, 68) = 34.44, *p* <.001, *η*^*2*^_*p*_ = 0.34, while there were no effects of group, *F*(2, 68) = 0.19, *p* =.831, or interactions between tasks and group, *F*(2, 68) = 1.45, *p* =.241. This suggests that performing the cognitive tasks increased anxiety levels uniformly across all groups.

**Fig. 2:**
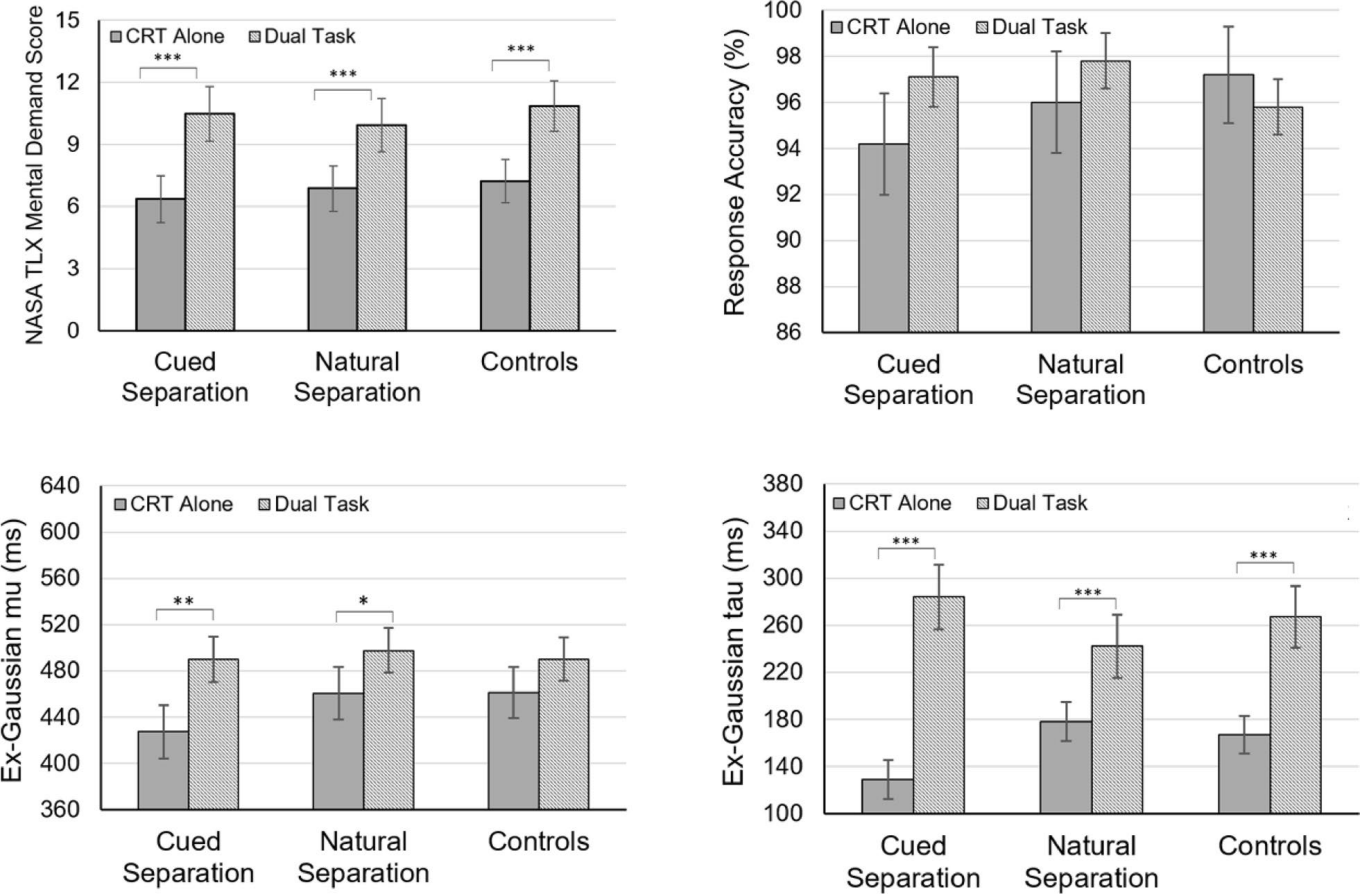
NASA Task Load Index (TLX) Mental Demand and Behavioral Performance among Groups. The error bars represent ± standard errors. *p <.05; **p <.01; ***p <.001

For perceived workload, repeated measures ANOVA indicated that performing both the CRT and the concurrent WM task significantly increased NASA TLX mental demand score compared to the CRT alone, *F*(1, 68) = 55.67, *p* <.001, *η*^*2*^_*p*_ = 0.44; however, there were no group differences in mental demand score, *F*(2, 68) = 0.12, *p* =.891, or in the effects of concurrent WM load on TLX mental demand score, *F*(2, 68) = 0.41, *p* =.667 (see Fig. 2).

### Effects of cellphone separation on cognitive performance

The effects of the cellphone separation groups and enhanced WM load on response accuracy and ex-Gaussian parameters were assessed through repeated measures ANOVA. The analysis of response accuracy revealed that neither concurrent WM load, *F*(1, 68) = 0.57, *p* =.451, nor group membership, *F*(2, 68) = 0.24, *p* =.785, significantly influenced CRT accuracy. Additionally, there was no interaction effect on accuracy, *F*(2, 68) = 0.66, *p* =.521 (see Fig. 2).

The repeated measures ANOVA for the ex-Gaussian parameter *μ* showed a significant main effect of WM load, *F*(1, 68) = 18.74, *p* <.001, *η*^*2*^_*p*_ = 0.22, but no effect of group, *F*(2, 68) = 0.40, *p* =.669. Furthermore, no interaction was found between the two factors, *F*(2, 68) = 1.01, *p* =.369 (see Fig. 2).

Ex-Gaussian parameter σ was not influenced by either WM load or group, and there were no interaction effects, *F*s < 3.11, *p*s >.083.

In contrast, the results for ex-Gaussian *τ* indicated no main effect of group, *F*(2, 68) = 0.02, *p* =.998; however, concurrent WM load significantly increased parameter *τ*, *F*(1, 68) = 79.72, *p* <.001, *η*^*2*^_*p*_ = 0.54, with an interaction effect present, *F*(2, 68) = 4.88,* p* =.010, *η*^*2*^_*p*_ = 0.16. Post hoc analyses revealed that WM load increased ex-Gaussian *τ* across all three groups, *t*s > 4.08, *p*s <.001, with the cued separation group exhibiting the largest effect of WM load on *τ* compared to the natural separation and control groups, *t*(69) = 2.89, *p* =.005. However, no significant differences were found in the effect of WM load on *τ* between the natural separation and control groups (see Fig. 2).

## Discussion

As cellphones and other mobile devices have become indispensable tools in technological societies, people are experiencing a variety of behavioral and health issues related to overuse and separation anxiety of those devices. The current study sought to address a potential mechanism through which cellphone separation may influence WM to impair EFs underlying emotion regulation and behavioral control. The dual task paradigm exhibited the effect of enhanced WM load on individuals’ cognitive performance at different mental representation activation levels of cellphone separation. We used a block design to deliver the cognitive tasks and recorded the self-report data for anxiety and perceived workload levels. Additionally, ex-Gaussian modeling was used to distinguish sensorimotor processes and top-down cognitive control.

Overall, our hypotheses were partially supported. Enhanced WM load by the concurrent memory task increased the levels of state anxiety and perceived workload while simultaneously reducing cognitive performance on the choice RT task. Importantly, while the three groups did not differ in the WM load effects on the ex-Gaussian parameters *μ* and *σ*, the cued separation group showed the greater effect of WM load on the parameter *τ* than did the two other groups. This finding is noteworthy as parameter *τ* presumes to infer top-down control processes. The current study was among the first to report the association between the level of representation activation of cellphone separation with precise cognitive effects of WM load.

The first hypothesis regarding the effects of WM load on task perception and performance was supported. In the dual task, the concurrent Sternberg memory task required participants to actively maintain the information in their WM and thus increased WM load, which was indicated by the higher rating scores of mental demand in NASA TLX. In turn, high WM load produced elevated levels of anxiety. The present findings are consistent with the positive relationship between perceived mental workload and negative affect (Yang et al., 2021, 2023, 2024). When tasks involve cognitive demands that exceed an individual’s available WM resources (i.e., cognitive stressors), the effortfulness of using EFs will induce subjective experience of negative emotions and reduce the individual’s motivation to maintain the same level of cognitive effort (Cacioppo & Petty, 1982).

The interaction between cognition and emotion is implicated in multitasking in everyday settings. For example, cognitive overload during driving gives rise to negative emotions and reduces the top-down control over affective processes, which increases the likelihood of operation errors in driving (Jeon & Zhang, 2013; Jeon et al., 2014; Sidoumou et al., 2015). Our findings of cognitive performance echoed the processes underlying the cognitive phenomena in these daily scenarios. Response speed (indicated by the ex-Gaussian parameter *μ*) was decreased and lapses in the controlled processes (indicated by the ex-Gaussian parameter *τ*) were increased by the concurrent load in the current study. However, in contrast to our prediction, response accuracy in the CRT task was not influenced by WM load. A ceiling effect may account for this null finding. Put plainly, the participants maintained high levels of accuracy in both blocks of the choice RT task, so it is possible that any cognitive effects of WM load on accuracy might be masked by these high accuracy scores. As a corollary of this account, there might be a speed-accuracy tradeoff among participants’ responses. Thus, future studies may use cognitive tasks with more complex response types to increase the difficulty of the primary task and the resultant WM load in the dual task paradigm. It could be possible that participants experienced higher levels of negative affect and cognitive stress in the second block compared to the early phase of the experiment, due to fatigue and boredom. However, we randomized the order of the block, so the present results are unlikely to be influenced by this confound.

Unlike our predictions in relation to WM load, the hypothesis regarding the group differences in the effects of WM load on subjective experience and performance on the cognitive task only received partial support of the results. The mental representation activation level of cellphone separation did not influence the effects of WM load on response accuracy. Due to the ceiling effect for response accuracy (see earlier sections), it is not surprising that none of the three groups’ accuracies were influenced by WM load. Interestingly, Eysenck et al.’s (2007) attentional control theory suggested that, if compensatory strategies are available, the quality of cognitive performance may not be impaired by anxiety, but processing efficiency may be reduced due to the recruitment of additional resources. This account is consistent with the present results that response accuracy (performance quality) remained unaffected, but response speed was prolonged by WM load and cellphone separation.

The ex-Gaussian parameter *μ* reflects automatic, low-level processing of RT stimuli (Spangler et al., 2018, 2021). Recall that the detrimental cognitive effects of cellphones are thought to be mediated by the impairments of EFs (Liebherr et al., 2020; Skowronek et al., 2023). Therefore, low-level sensorimotor processing, contrasting the high-level processing of EFs, does not seem subject to the impacts of WM load. Although the null results of ex-Gaussian *μ* were indeterminate, they may help explain the mixed findings in the research on distraction caused by cellphones (e.g., Hartmann et al., 2020; Ruiz Pardo & Minda, 2022) and are expected to be investigated in future studies.

Contrarily, our results demonstrate that the prolongation of the ex-Gaussian *τ* was largest in the cued separation group compared to other groups. According to Cohen’s (1988) criteria, the effect size of the interaction was considered as a large effect (partial eta squared > 0.14). In these results, the interaction between group and task condition might be driven be shorter tau in the CRT alone condition in the cued separation group, compared to two other groups. However, a closer examination on group effects on raw scores of ex-Gaussian parameters indicated no group differences in the ex-Gaussian *τ* (see Table 1). Therefore, it would be proper to interpret the interaction as the effect of cellphone separation on tau prolongation by WM load. In the cued separation group, a cue (e.g., the cellphone did not belong to the participant) was presented to activate the mental representation of the participant’s own cellphone. As a result, WM was impaired by not only the fake cellphone but cellphone separation as well, whereas the two other groups did not receive the same level of WM interference. This interpretation is consistent with the previous findings (Hartanto & Yang; Ward et al., 2017), which point that deteriorating effect of cellphone on cognition might be explained by mental representations of distraction and/or a reward cue of information access or social connection. Those associations of cellphones with distraction and rewards are likely established from frequent use of cellphones (Hartanto & Yang, 2016; Ward et al., 2017). Yet, the effects of WM load on anxiety level were not modulated by group. The inconsistency of patterns between groups suggests that the depletion of WM resources might occur subliminally, only implicitly impacting performance and not influencing subjective experiences during tasks. Another possible contributing factor is that the visual presentation of the fake cellphone would increase the cognitive load at the perceptual level, which enlarged the effect of WM load on CRT performance. Although this factor is not contradictory to our interpretation of the results, high fidelity simulation (e.g., virtual reality) may be used to control and test visual attention in cognitive performance.

It is worth noting that the effects of cellphones on cognition may be unspecific. The performance on the Sternberg WM task (the secondary task) was not different among the groups. These null results are likely to indicate that the specific cognitive resources required by the secondary task was not influenced by the mental representation of cellphones. However, without data of other dimensions (e.g., physiological and neural imaging data), this possibility could not be confirmed in the present study. Additionally, the ex-Gaussian *τ* can serve as an indicator of the overall level of cognitive resources being used in a continuous goal-directed process (e.g., sustained attention; Spangler et al., 2018, 2021). In that regard, the present results suggest that the presence, availability, and separation of cellphones may impair the supervisory attention system (Baddley, 1986).

### Implications

The present study has several practical implications. In educational settings, the integration of mobile technologies in teaching and learning is quickly expanding and the influences of cellphones, as well as other devices, on teaching and learning may soon become unavoidable. Simply forbidding the use of cellphones may induce anxiety along with causing implicit impairments for ongoing cognitive activities. Give the nonspecific nature of the cognitive effects of cellphones, the methods to eliminate undesirable effects may include controlling the length of material delivery in classrooms and an enhancement of course content to compete for cognitive resources with the cellphone-related representations. In transportation and the workplace, human errors may be reduced by replacing certain functions of cellphones, such as communication and information acquisition with other devices that are exclusively designed for the primary task (e.g., driving and manufacturing operations). These approaches will reduce the reliance on cellphones and deactivate the mental representation of cellphones. Additionally, as Hartanto et al. (2024) summarized in a recent review, complete removal of smartphones from a work environment would not improve productivity and performance. Our findings suggested that the key to reduce the negative effects of smart device is to deactivate its mental representation as well as its associations with rewards of social connection and information access.

### Limitations, future studies, and conclusion

Our findings need to be evaluated in the light of a few limitations. First, the sample size was relatively small. We used the parameters from the previous studies using similar experimental designs in the priori power analysis. However, due to the lack of studies on cellphone separation and the ex-Gaussian modeling, the sample size might be estimated as too small, which raises potential concerns about replicability. In future studies, a more conservative power analysis should be conducted to replicate our findings. Second, there were no direct measures of nomophobia in the present study. We only included the scale to assess unspecific state anxiety and there was no group effect on anxiety elevation during the task. It is important to directly measure nomophobia to disentangle affective and non-affective factors in cellphone separation in future studies. Similarly, there were no direct measurements of the brain areas that are involved in WM. Physiological and neural recordings should be included in future studies. Next, we had only two WM load levels, which limits the validity of our findings. In applied settings, mental workload may vary at multiple levels or as a continuous parameter, comparisons of more WM load levels will clarify the quantitative relationship of cellphones with cognitive performance. Fourth, our sample predominantly consisted of female participants. Given the gender differences in technology usage and reliance, gender-balanced samples should be included in future studies. Moreover, gender differences in the effects of cellphone separation should be studied in the future for a comprehensive understanding of cellphone separation effects on WM functions between users. Last but not least, the relationships between NASA-TLX subscale scores and other tools to assess workload should be studied and clarified in future studies of cellphone separation.

Despite these limitations, our study utilized direct manipulations on the mental representation activation levels of cellphones and enhanced WM load. Also, ex-Gaussian modeling was used to derive separate indices of automatic and controlled cognitive processes, respectively. Therefore, the positive features of our study certainly outweighed its drawbacks, and our findings add to the emerging literature on the interaction between humans and mobile technologies.

## Data Availability

The raw data supporting the conclusions of this article will be made available by the authors on request.
